# Candidate Circulating microRNAs in Patients with Sarcopenic Obesity: Results of a Pilot Screening

**DOI:** 10.3390/biomedicines14061377

**Published:** 2026-06-18

**Authors:** Nela Chobolová, Zdeněk Švagera, David Stejskal, Marek Bužga

**Affiliations:** 1 Institute of Laboratory Medicine, Faculty of Medicine, University of Ostrava, 70300 Ostrava, Czech Republic; nela.chobolova@fno.cz (N.C.); zdenek.svagera@fno.cz (Z.Š.); david.stejskal@fno.cz (D.S.); 2 Institute of Laboratory Medicine, Department of Clinical Biochemistry, University Hospital Ostrava, 70852 Ostrava, Czech Republic; 3 Department of Physiology and Pathophysiology, Faculty of Medicine, University of Ostrava, 70300 Ostrava, Czech Republic

**Keywords:** sarcopenic obesity, biomarkers, circulating microRNAs, RT-qPCR, muscle–adipose crosstalk, muscle atrophy, metabolic dysfunction, pilot study

## Abstract

**Background/Objectives**: Sarcopenic obesity (SO) represents a severe clinical phenotype characterized by the coexistence of reduced skeletal muscle mass and excess adiposity, and is associated with insulin resistance, dyslipidemia, and systemic inflammation. However, easily accessible biomarkers that capture early molecular changes underlying SO are lacking. The aim of this pilot study was to compare circulating microRNA (miRNA) profiles in patients with severe obesity and a sarcopenic obesity phenotype with those of healthy controls and to identify candidate miRNAs suitable for further validation. To the best of our knowledge, this represents one of the first broad screening studies of circulating miRNAs specifically conducted in patients with severe obesity and DXA-confirmed sarcopenic obesity. **Methods**: In this single-center pilot study conducted in the Czech Republic, fasting plasma samples from 12 adult participants (6 with severe obesity and sarcopenic obesity phenotype, body mass index > 45 kg/m^2^; 6 healthy controls; age 18–65 years) were analyzed using an RT-qPCR panel comprising 384 assays, including technical controls and 352 target circulating miRNAs. Following predefined quality control and filtering criteria, 224 miRNAs were retained for the final statistical analysis. Six patients with severe obesity were classified according to the ESPEN/EASO 2022 consensus criteria for sarcopenic obesity, while EWGSOP2-based assessment was used for functional evaluation of sarcopenia. Differential expression was evaluated using fold change and exploratory statistical testing. **Results**: We identified a set of miRNAs with significantly altered expression in SO, including increased muscle-enriched miR-486-5p and hepatocyte-enriched miR-122-5p, and decreased vascular miR-145-5p, as well as several additional miRNAs related to myogenesis, lipid metabolism and inflammatory signaling. miR-451a, a recognized marker of hemolysis, was also increased but was interpreted with caution. **Conclusions**: Despite the limited sample size, the results of this study suggest that specific circulating miRNAs may reflect key pathophysiological pathways in SO and could serve as promising biomarkers to support risk stratification and monitoring in larger, hypothesis-driven studies.

## 1. Introduction

Obesity represents one of the most significant public health challenges worldwide and is associated with increased morbidity, mortality, and healthcare costs [1,2]. In Central and Eastern Europe, the prevalence of obesity in the adult population continues to rise, and a substantial proportion of patients develop severe obesity with multiple cardiometabolic complications [1,2]. Bariatric and metabolic surgery has become the most effective intervention for long-term weight reduction and improvement in metabolic parameters, including glycemic control and lipid profile [3,4,5]. However, it should not be regarded solely as a physical intervention, as the persistence of inflammatory cellular memory and sustained activation of pro-inflammatory pathways may continue to contribute to metabolic and inflammatory dysregulation even after significant weight loss. This concept is increasingly recognized in the literature and highlights the complex, multifactorial nature of obesity and its associated complications.

Despite these therapeutic options, a proportion of patients remain at high cardiometabolic risk even after weight reduction. In particular, sarcopenic obesity (SO) is increasingly recognized as a distinct and clinically relevant phenotype [5]. According to the revised European Working Group on Sarcopenia in Older People 2 (EWGSOP2) consensus, sarcopenia is defined primarily by low muscle strength, confirmed by reduced muscle quantity or quality, and in advanced stages, accompanied by impaired physical performance [6]. However, EWGSOP2 criteria were primarily developed for the diagnosis of sarcopenia, particularly in older adults, and may be limited in individuals with severe obesity, in whom absolute muscle mass can remain relatively preserved. Therefore, for the classification of sarcopenic obesity in the present cohort, the ESPEN/EASO 2022 consensus framework was applied, as it specifically addresses the coexistence of excess adiposity and impaired muscle status [7]. In the context of obesity, the coexistence of excess adiposity with reduced muscle strength and mass represents a particularly adverse phenotype, commonly referred to as sarcopenic obesity [8,9].

The reported prevalence of sarcopenic obesity in adults varies widely depending on the diagnostic criteria and population studied, generally ranging from approximately 5% to over 20%, with higher rates observed in older individuals and in those with severe obesity [8,9]. Major risk factors include aging, physical inactivity, chronic low-grade inflammation, insulin resistance, hormonal dysregulation, inadequate protein intake, and metabolic disturbances associated with excess adiposity [6,8,9,10,11]. SO is associated with decreased physical performance, frailty, and a higher incidence of cardiovascular and metabolic complications driven by obesity, insulin resistance, and chronic inflammation [6,8].

In clinical practice, the diagnosis of sarcopenic obesity in adults is based on the combined assessment of muscle strength, muscle mass, and physical performance, most commonly using handgrip strength testing together with body composition analysis by DXA or BIA, according to established consensus criteria [6,12]. However, these methods primarily reflect structural and functional impairment and may not fully capture early molecular alterations preceding measurable decline.

Current management strategies focus on multimodal interventions, including resistance exercise training, optimization of protein intake, weight-reduction strategies, and treatment of metabolic comorbidities. Despite these approaches, no specific pharmacological therapy for sarcopenic obesity has yet been established, and sensitive molecular biomarkers supporting early diagnosis, risk stratification, and personalized treatment remain lacking.

Although numerous circulating and molecular biomarkers have been investigated in relation to sarcopenia and sarcopenic obesity in adult populations, including inflammatory markers, hormonal parameters, and composite biochemical indices, none has yet achieved sufficient validation, standardization, or consensus for routine clinical implementation [12,13,14,15,16,17,18,19,20,21]. Recent studies have further explored emerging biomarker candidates and multi-parameter diagnostic approaches, highlighting both the biological complexity of sarcopenic obesity and the persistent lack of universally accepted diagnostic tools [12,20,21]. Proposed markers, such as the creatinine-to-cystatin C ratio, inflammatory cytokines and myokines, or imaging-based indices, provide only partial information and often reflect relatively late stages of the disease [8,13,14]. Conventional biochemical and anthropometric parameters, therefore, offer limited insight into early molecular changes in muscle and adipose tissue and into the dynamic nature of metabolic remodeling [16,17,18,19].

Importantly, sarcopenic obesity should not be regarded merely as the coexistence of two conditions, but rather as a distinct pathophysiological entity characterized by disrupted inter-organ communication between skeletal muscle, adipose tissue, and metabolic organs [9,20,22,23]. Its development involves chronic low-grade inflammation, insulin resistance, anabolic resistance of skeletal muscle, mitochondrial dysfunction, and altered myokine–adipokine signaling [6,9,20,22,23,24]. These interconnected mechanisms generate a complex metabolic environment that cannot be adequately reflected by isolated laboratory markers. This mechanistic complexity represents a critical gap in current biomarker research, as most available markers assess isolated metabolic or inflammatory components rather than integrated multiorgan dysfunction [12,20,21].

miRNAs have emerged as promising candidates for minimally invasive biomarkers in this context. These small non-coding RNAs regulate gene expression post-transcriptionally and participate in pathways relevant to muscle growth, atrophy, adipogenesis, insulin signaling, and inflammation [9,25,26,27,28,29,30,31].

Several studies have investigated circulating miRNAs in sarcopenia, obesity, and related metabolic disorders. MyomiRs such as miR-486-5p and miR-206 have been shown to regulate myogenesis via the IGF-1/PI3K/AKT pathway and are responsive to physical activity and muscle adaptation [9,22,29,32,33,34]. miR-122-5p, a hepatocyte-enriched miRNA, is consistently elevated in obesity, non-alcoholic fatty liver disease (NAFLD), and metabolic syndrome, reflecting hepatic lipid dysregulation and metabolic burden [22,35,36,37,38,39]. In contrast, miR-145-5p, which contributes to vascular smooth muscle homeostasis, is reduced in settings of endothelial dysfunction and cardiometabolic disease [22,33,40]. Metabolic miRNAs, including miR-33, miR-378, and members of the miR-30 family, have been implicated in cholesterol homeostasis, adipogenesis, and triglyceride metabolism [41,42,43,44,45,46].

Furthermore, exercise-responsive miRNAs, including miR-1, miR-133a, and miR-206, are modulated by resistance and aerobic training and have been proposed as dynamic indicators of muscle adaptation and metabolic remodeling [47,48,49,50,51,52,53,54,55]. However, the specific pattern of circulating miRNAs characterizing patients with confirmed sarcopenic obesity, defined by the concurrent presence of excess adiposity and reduced relative muscle mass according to established consensus criteria, remains insufficiently characterized. Existing studies have typically addressed either sarcopenia or obesity in isolation or have focused on targeted panels limited to a small number of predefined miRNAs [9,22,26,47]. This represents an important knowledge gap, as sarcopenic obesity involves a distinct and complex interaction between skeletal muscle, adipose tissue, and metabolic organs that may not be adequately captured by studies focusing on sarcopenia or obesity separately.

Therefore, the aim of this pilot study was to perform a broad screening of circulating miRNAs in patients with severe obesity and sarcopenic obesity phenotype compared with healthy controls. Unlike hypothesis-driven studies focusing on a limited number of predefined targets, this approach enabled the simultaneous evaluation of a large panel of miRNAs, facilitating the identification of novel candidate biomarkers and providing a more comprehensive insight into the molecular landscape associated with sarcopenic obesity. The identified candidate miRNAs will be further validated in larger, hypothesis-driven studies. Thus, this high-throughput screening approach represents a key strength of the study despite the limited sample size.

Therefore, this study was designed as a pilot, hypothesis-generating screening study rather than a confirmatory analysis.

## 2. Materials and Methods

### 2.1. Study Population

A total of 12 plasma samples were included in this pilot study: 6 healthy controls and 6 patients with severe obesity (BMI > 45 kg/m^2^) and sarcopenia. The study involving healthy controls was approved by the Ethics Committee of the Faculty of Medicine, University of Ostrava (SGS06/LF/2025). Baseline characteristics of the study participants are summarized in Table 1.

The severe obesity group was characterized by extreme obesity, with a mean BMI of 51.1 ± 8.8 kg/m^2^. Compared with healthy controls, participants in the severe obesity group showed lower mean handgrip strength. DXA-derived body composition analysis demonstrated markedly elevated adiposity parameters in the severe obesity group, including total body fat percentage and visceral adipose tissue (VAT) area, together with lower ALM/W values. Despite severe obesity, absolute ALMI values remained relatively preserved in the study cohort, highlighting the heterogeneous phenotype of sarcopenic obesity. This pilot study was nested within the ongoing SarxOb randomized controlled trial (NCT04617392), which evaluates the effects of bariatric surgery and structured exercise on sarcopenic obesity. Patients referred to the Bariatric Surgery Center of Hospital AGEL Ostrava-Vítkovice for laparoscopic sleeve gastrectomy were screened by the multidisciplinary bariatric team during pre-operative outpatient visits. Patients fulfilling all inclusion criteria for sarcopenic obesity and none of the exclusion criteria were invited to participate in the SarxOb trial and received written and oral information about the study. Those willing to participate provided written informed consent before any study-specific procedures were performed.

For the present miRNA substudy, we used baseline fasting plasma samples collected at the pre-operative visit (≤45 days before surgery). At the time of sample selection, a total of 56 participants had been enrolled in the parent SarxOb study. All enrolled patients had a sufficient number of EDTA plasma aliquots available. Subsequently, from the available plasma aliquots, six participants from all SarxOb participants with confirmed sarcopenic obesity and severe obesity (BMI > 45 kg/m^2^) were selected for miRNA screening using simple random sampling based on a computer-generated random sequence.

The control group consisted of 6 healthy volunteers aged 18–65 years with BMI < 25 kg/m^2^, without chronic diseases or medication affecting muscle or adipose tissue metabolism. Controls were recruited from the hospital and university community through local advertisements and word of mouth and were screened for medical history and basic laboratory tests to confirm eligibility.

This sample size represents the minimum number required to ensure basic analytical reliability for exploratory comparisons between diagnostic groups. Due to the exploratory pilot design, a formal sample size calculation was not performed. The sample size corresponds to a pilot screening primarily intended to detect large effect sizes.

Biological material from patients included in this pilot study was collected as part of the project No. NW24-09-00016: Skeletal Muscle as an Endocrine Organ: The Role of Myokines in Muscle Metabolism and Other Metabolic Organs. The parent study was approved by the Ethics Committee of AGEL Hospital Ostrava–Vítkovice (EK/125/2022).

### 2.2. Clinical and Laboratory Data Collection

All study procedures were performed according to the SarxOb study protocol. At the baseline visit (≤45 days before surgery for SO patients and at enrolment for controls), a trained study nurse obtained a detailed medical history, including comorbidities and current medication, and measured anthropometric parameters (body weight, height, waist circumference) using calibrated equipment. Body mass index (BMI) was calculated as weight divided by height squared. In both study groups, body composition was assessed by dual-energy X-ray absorptiometry (DXA) using a Hologic Horizon A DXA system (S/N 301514M; software version 13.6.0.5; Hologic Inc., Marlborough, MA, USA). The DXA system used fan-beam technology and NHANES BCA calibration. In the severe obesity group, these measurements were used to classify sarcopenic obesity according to the ESPEN/EASO 2022 criteria [7]. Muscle strength was additionally assessed by handgrip dynamometry (KERN & SOHN GmbH, Balingen, Germany) in the dominant hand in a seated position with the elbow flexed at 90°, in accordance with the EWGSOP2 protocol [6]. Physical performance was assessed using the 6 m gait speed test. Both assessments were performed by trained study personnel using standardized protocols at the pre-operative baseline visit.

The same preanalytical protocol was applied to both the severe obesity group and healthy controls. Anthropometric measurements and handgrip strength assessment were performed according to the same standardized protocol in both groups. After at least a 12 h overnight fast, venous blood was drawn in the morning from the antecubital vein into EDTA tubes while the participant was seated, according to a standardized protocol. Blood collection and sample handling were performed by trained healthcare personnel using identical procedures in both groups. Subsequent sample processing and storage procedures are described in Section 2.4.

### 2.3. Inclusion and Exclusion Criteria

The original project includes patients with sarcopenic obesity (SO) who meet several key inclusion criteria. Eligible individuals are 18–65 years old, with BMI ≥ 35 kg/m^2^ in the presence of comorbidities, or BMI ≥ 40 kg/m^2^ regardless of associated diseases. The diagnosis of sarcopenic obesity was based on the ESPEN and EASO 2022 consensus criteria [7], which specifically address the diagnostic challenge in obesity by requiring excess adiposity together with reduced appendicular lean mass adjusted for body weight (ALM/W), rather than absolute lean mass indexed to height squared. Reduced skeletal muscle mass was defined as ALM/W below sex-specific reference thresholds (<23.47% in women and <28.27% in men) derived from Weber et al. [11] and applied within the ESPEN/EASO 2022 diagnostic framework [7]. Excess adiposity was defined as a total body fat percentage > 38% in women and >30% in men, as assessed by DXA [7]. Five of six patients fulfilled both criteria; one patient (SX60P) had borderline ALM/W (24.1%) with marked excess adiposity (46.6%) and was included based on the primary screening criterion (BMI > 40 kg/m^2^). Another requirement for inclusion is a clinical indication for bariatric surgery according to standard clinical guidelines. Patients must also be able to understand the study conditions and sign informed consent.

Exclusion criteria include active malignant disease, severe chronic inflammatory or autoimmune disorders, renal or hepatic failure, use of medication significantly affecting muscle or adipose tissue metabolism (e.g., high-dose corticosteroids), pregnancy, and breastfeeding.

The control group consisted of healthy volunteers with a BMI up to 25 kg/m^2^, without comorbidities or medications affecting metabolism.

### 2.4. Sample Collection and Handling

Blood samples were collected into ethylenediaminetetraacetic acid (EDTA) tubes. Immediately after collection, samples were centrifuged (1500 *g*, 10 min, 4 °C), and plasma aliquots were promptly stored at −80 °C until analysis without repeated freeze–thaw cycles. To minimize preanalytical variability, all samples were handled under standardized conditions. A dedicated laboratory hemolysis index was not available for this pilot set. Therefore, potential hemolysis was assessed indirectly during data interpretation using miR-451a, a well-recognized erythrocyte-enriched miRNA. Accordingly, findings involving miR-451a were interpreted with caution.

### 2.5. RNA Isolation and miRNA Profiling

RNA was isolated using the iCatcher Circulating cfRNA 1000 Kit (Cat. No. AC20100-36, CatchGene, Guangzhou, China) from 1 mL of plasma and analyzed by RT-qPCR using the MIRXES ID3EAL miRNA Knowledge Panel 384 Targets (Cat. No. 1105263, MiRXES, Singapore). A total of 352 target miRNAs were assessed. The panel was selected by the manufacturer based on extensive internal research data and published literature.

### 2.6. Data Processing and Normalization

To normalize technical variability, an RT spike-in control and an inter-plate control were used (according to the manufacturer’s instructions). To correct for biological variability, normalization to the global mean expression of all quantified miRNAs was applied.

### 2.7. Statistical Analysis and Data Evaluation

Statistical analysis was performed using a predefined evaluation template provided by the assay manufacturer (MIRXES), with data processing implemented in R (version 4.2.3; R Foundation for Statistical Computing, Vienna, Austria). The volcano plot was generated using the ggplot2 package (version 3.4.0) in R.

To reduce technical variability, data were normalized using RT spike-in and inter-plate controls. To account for biological variability between samples, normalization to the global mean expression of all detected miRNAs was applied. Only miRNAs detected above the limit of detection and present in at least three samples within one biological group were included in the statistical analysis.

Group comparisons were conducted using Welch’s *t*-test, which was selected due to the small sample size and the assumption of unequal variances between groups. Normality of data distribution was assessed using the Shapiro–Wilk test. Due to the limited sample size, results of normality testing were interpreted cautiously and are provided in Supplementary Table S3.

Given the exploratory screening design of the study and the limited sample size, *p*-values were interpreted as indicative rather than confirmatory. No correction for multiple testing was applied in order to reduce the risk of false-negative findings and to prioritize candidate miRNAs for further validation. This approach is consistent with the exploratory and hypothesis-generating nature of pilot miRNA screening studies, in which identification of potentially biologically relevant candidates is prioritized over strict control of false-positive findings.

Relative changes in expression between groups were evaluated and expressed as fold change and log_2_ fold change. A fold-change threshold of |log_2_FC| > 1.5 was applied as a criterion for biological relevance; statistical significance was assessed separately using *p*-values from Welch’s *t*-test. Findings meeting both criteria (|log_2_FC| > 1.5 and *p* < 0.05) were considered candidate miRNAs for further validation.

**Figure 1:**
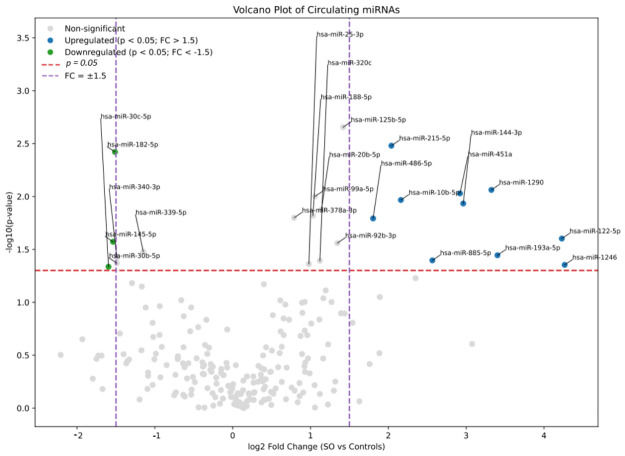
Volcano plot of circulating miRNAs in SO vs. controls (each dot represents one miRNA).

To ensure transparency and reproducibility, complete datasets are provided in the Supplementary Materials, including Supplementary Table S1 (primary raw Cq data) and Supplementary Table S2 (processed miRNA dataset with expression values, fold changes, and statistical parameters). The volcano plot presented in Figure 1 was generated from the complete processed dataset containing all statistically evaluated miRNAs.

## 3. Results

### 3.1. Detection and Filtering of Circulating miRNAs

Although the panel comprised 352 miRNAs, only 224 met the predefined detection criteria and were included in the final analysis. These 224 targets were defined as miRNAs detected above the limit of detection and reliably identified in at least three samples within each biological group.

### 3.2. Differential Expression of Candidate miRNAs

The analysis revealed several significantly deregulated miRNAs in patients with severe obesity and sarcopenia. Downregulated miRNAs included miR-30c-5p (*p* = 0.046), miR-145-5p (*p* = 0.027), and miR-182-5p (*p* = 0.004). Conversely, the following miRNAs were upregulated: miR-486-5p (*p* = 0.016), miR-215-5p (*p* = 0.003), miR-10b-5p (*p* = 0.011), miR-885-5p (*p* = 0.040), miR-144-3p (*p* = 0.012), miR-1290 (*p* = 0.009), miR-193a-5p (*p* = 0.036), miR-122-5p (*p* = 0.025), and miR-1246 (*p* = 0.044). Although miR-451a also showed increased expression, it is a known marker of hemolysis and therefore is not recommended for further validation.

Table 2 includes all miRNAs that meet the predefined threshold for biological relevance (|log_2_FC| > 1.5), irrespective of statistical significance. Candidate miRNAs additionally meeting the criterion of *p* < 0.05 are marked with an asterisk and were considered primary candidates for further validation. The most promising candidates were identified based on a combination of a large expression change (high absolute log_2_FC) and statistical significance. For significantly deregulated candidate miRNAs, data distribution was additionally assessed using the Shapiro–Wilk test and did not show major deviations from normality (Supplementary Table S3).

A complete overview of all evaluated miRNAs, including those not meeting statistical significance, is provided in the Supplementary Materials together with the underlying raw Cq data for individual samples.

Data are presented as mean ± standard deviation (SD); SO, sarcopenic obesity; Cq, quantification cycle; *p*-values were calculated using Welch’s *t*-test (SO vs. controls). Due to the exploratory design and limited sample size, *p*-values should be interpreted with caution.

The results are graphically illustrated in Figure 1 (Volcano plot), which displays the differential expression of individual miRNAs between patients with sarcopenic obesity and healthy controls. The volcano plot was generated using the complete dataset of all statistically evaluated miRNAs (*n* = 224), as provided in the Supplementary Materials. The horizontal axis represents the log_2_ fold change (the logarithmically expressed ratio of expression change between the two groups), while the vertical axis shows −log_10_ (*p*-value), representing the logarithmically expressed measure of statistical significance.

Blue (upregulated) and green (downregulated) data points indicate statistically significant dysregulated miRNAs (*p* < 0.05), whereas yellow points correspond to non-significant changes in expression (color coding is for visualization purposes only).

### 3.3. Functional Annotation of Identified Candidate miRNAs

The functional annotation summarized in Table 3 was performed based on a comprehensive review of published literature for each individual candidate miRNA. The biological functions and relevance to sarcopenic obesity presented in Table 3 are further discussed and supported by literature references in Section 4.1.

A functional annotation of the selected candidate miRNAs is summarized in Table 3, which provides an overview of their known biological roles and direction of expression changes observed in the present study.

## 4. Discussion

Sarcopenic obesity represents a complex and multifactorial phenotype characterized not only by the coexistence of excess adiposity and reduced muscle mass but also by profound metabolic and inflammatory dysregulation. Chronic low-grade inflammation, insulin resistance, and altered inter-organ communication between skeletal muscle and adipose tissue are considered key mechanisms contributing to its development and progression.

In this context, circulating microRNAs may reflect underlying molecular alterations associated with these processes. The dysregulated miRNAs identified in this study may be linked to pathways involved in muscle remodeling, lipid metabolism, and inflammatory signaling, thereby providing insight into the biological mechanisms underlying sarcopenic obesity.

Several studies have shown that exercise interventions, nutritional strategies, and bariatric surgery can modulate circulating miRNAs involved in myogenesis, inflammation, and metabolic regulation, suggesting their potential utility as dynamic biomarkers of treatment response [35,47,48,51,52,53,56]. Given their tissue-specific or tissue-enriched origin and targeted regulation of key signaling cascades, miRNAs can reflect both structural and functional changes in muscle and metabolic organs [9,22,29,30,32,57,58]. In addition to their diagnostic value, several miRNAs have been proposed as therapeutic targets or mediators of adaptive responses to exercise and nutritional interventions.

Profiling circulating miRNAs, therefore, represents a promising approach for capturing the complex interplay between skeletal muscle, liver, and adipose tissue in sarcopenic obesity. Such profiles may support earlier diagnosis, risk stratification, prediction of treatment response, and personalized monitoring of patients over time.

### 4.1. Biological and Clinical Interpretation of Candidate miRNAs

Several key circulating miRNAs were identified in the study cohort as potentially relevant to the pathophysiology of sarcopenic obesity. Current evidence suggests that miRNAs participate in the complex crosstalk between skeletal muscle, adipose tissue, liver, and the cardiovascular system, thereby contributing to the metabolic and inflammatory disturbances characteristic of sarcopenic obesity [9,22,29,57,58]. Among the most prominent were miR-486-5p, miR-122-5p, miR-30c-5p, and miR-182-5p, representing muscle-, liver-, and metabolism-associated pathways. In addition, miR-145-5p and selected less tissue-specific miRNAs (e.g., miR-1290 and miR-1246) may reflect vascular and systemic metabolic alterations. Together, these miRNAs capture key molecular features of sarcopenic obesity, linking muscle remodeling, lipid metabolism, inflammation, and inter-organ communication.

The present pilot study adds to the existing body of evidence by focusing specifically on patients with severe obesity fulfilling the SarxOb study criteria for sarcopenic obesity. Using a screening panel of circulating miRNAs, we identified a set of molecules with altered expression that may reflect key aspects of the pathophysiology of SO and may serve as suitable biomarker candidates.

miR-486-5p (log_2_FC = 1.80; *p* = 0.016) was significantly upregulated in the study cohort. This miRNA is strongly enriched in skeletal muscle and is induced by physical activity [33]. It belongs to the group of myomiRs that promote myogenesis through activation of the IGF-1/PI3K/AKT pathway and inhibition of PTEN/FOXO1A signaling, thereby supporting muscle growth and regeneration. Beyond its mechanistic role, miR-486-5p has been proposed as a potential circulating biomarker for muscle wasting conditions. Reduced plasma levels have been reported in patients with Duchenne muscular dystrophy and in aging-related muscle loss, suggesting its diagnostic value for conditions of impaired muscle integrity [9,22,29,32]. The observed upregulation, therefore, supports its potential utility as a circulating biomarker reflecting altered muscle remodeling in sarcopenic obesity.

miR-145-5p (log_2_FC = −1.54; *p* = 0.027), which was decreased in patients with SO, is involved in the differentiation of vascular smooth muscle cells and maintenance of vascular homeostasis [40]. Reduced expression has been associated with endothelial dysfunction, impaired vasomotor regulation, and increased arterial stiffness, all of which are common features in obesity-related cardiometabolic disease. Reduced circulating miR-145-5p has been associated with endothelial dysfunction and impaired vascular homeostasis in cardiometabolic disease, supporting its potential utility as a prognostic marker of vascular complications in obesity-related conditions [22,33]. The decreased expression observed in the present study may therefore reflect vascular impairment accompanying sarcopenic obesity and further supports its relevance as a candidate biomarker of obesity-related cardiometabolic dysfunction.

miR-122-5p (log_2_FC = 4.23; *p* = 0.025) was significantly increased in patients with sarcopenic obesity. This liver-enriched miRNA is a key regulator of hepatic lipid metabolism and is recognized as a biomarker of non-alcoholic fatty liver disease and metabolic syndrome [22,35,37,38,39]. Elevated circulating levels may therefore reflect not only hepatic fat accumulation and dyslipidemia, but also the broader metabolic burden associated with concurrent muscle loss and excessive adiposity. Circulating miR-122-5p has been extensively validated as a biomarker of hepatocellular damage and metabolic liver disease, demonstrating high sensitivity for NAFLD and hepatic inflammation [37,38,39]. Elevated levels observed in patients with sarcopenic obesity are therefore not only mechanistically plausible but also diagnostically informative as a potential marker of hepatic metabolic burden accompanying sarcopenic obesity [9,22,35,37,38,39]. Its marked elevation further supports its potential use as a biomarker of obesity-associated hepatic and metabolic dysfunction in sarcopenic obesity.

Both miR-30c-5p (log_2_FC = −1.60; *p* = 0.046) and miR-182-5p (log_2_FC = −1.51; *p* = 0.004) were decreased in the study cohort. miR-30c-5p participates in the regulation of lipoprotein secretion and triglyceride metabolism, and its downregulation has been associated with hypertriglyceridemia and impaired lipid clearance [44,45,46,59,60]. miR-182-5p has been linked to oxidative stress responses and cellular adaptation to metabolic stress through regulation of NOX4-dependent signaling pathways. Reduced expression may increase cellular susceptibility to oxidative injury and impair stress-adaptive responses [61]. Lower circulating miR-30c-5p has been linked to hypertriglyceridemia and impaired lipid clearance and has been proposed as a potential biomarker of dyslipidemia in metabolic syndrome, consistent with the metabolic profile of patients with sarcopenic obesity [32,45,46,57,59].

miR-1290 (log_2_FC = 3.32; *p* = 0.009) and miR-1246 (log_2_FC = 4.39; *p* = 0.044), although less tissue-specific, have been associated with adverse metabolic status, systemic inflammation, and metabolic dysregulation [9,22,29,54,62,63]. Their increased expression may reflect obesity-related metabolic stress and inflammatory signaling. In combination with muscle-enriched and hepatic miRNAs, they may help capture the multidimensional pathophysiology of sarcopenic obesity.

Among these candidates, miR-1290 has previously been reported to be elevated in patients with obesity and diabetes, suggesting its potential utility as a circulating biomarker of metabolic dysfunction and insulin resistance [62]. Recent evidence further indicates that miR-1246 participates in adipose tissue homeostasis and exosome-mediated intercellular communication through modulation of macrophage polarization and adipose tissue remodeling, processes closely linked to obesity-associated inflammation and metabolic regulation [63]. Although its biomarker potential in sarcopenic obesity remains to be established, the increased expression observed in the present study may reflect altered adipose tissue signaling and systemic metabolic adaptation associated with obesity-related metabolic dysfunction [54,62,63].

miR-193a-5p (log_2_FC = 3.40; *p* = 0.036), miR-885-5p (log_2_FC = 2.57; *p* = 0.040), and miR-144-3p (log_2_FC = 2.96; *p* = 0.012) were also elevated and have previously been implicated in the regulation of cell proliferation, lipid metabolism, and oxidative stress [9,17,22,26,57,60,64,65,66,67,68]. Although their specific roles in SO remain unclear, the results of this study suggest that they may contribute to fine-tuning of metabolic and inflammatory signaling in muscle and adipose tissue. Larger cohort studies are needed to elucidate their biological relevance. Recent evidence has identified circulating miR-193a-5p as a biomarker associated with the progression of non-alcoholic fatty liver disease (NAFLD), supporting its potential relevance in obesity-related metabolic dysregulation and hepatic dysfunction [69]. Given the close relationship between sarcopenic obesity, insulin resistance, and ectopic fat accumulation, the increased expression observed in the present study may reflect broader metabolic disturbances extending beyond skeletal muscle and adipose tissue. Circulating miR-885-5p has been proposed as a biomarker of liver pathology and metabolic dysregulation [65]. Its elevated expression in the present cohort may therefore reflect the systemic metabolic burden associated with sarcopenic obesity. miR-144-3p has been implicated in adipogenesis and metabolic regulation through targeting of the FOXO1 signaling pathway [67]. In addition, adipose-derived miR-144-3p has been suggested as a potential biomarker of obesity-related metabolic alterations [68]. However, because miR-144-3p is enriched in erythrocytes, interpretation of its circulating levels should be approached cautiously in the absence of a formal hemolysis assessment.

Additional miRNAs potentially reflecting metabolic and inflammatory changes in SO included miR-215-5p (log_2_FC = 2.04; *p* = 0.003), miR-10b-5p (log_2_FC = 2.16; *p* = 0.011), and miR-451a (log_2_FC = 2.92; *p* = 0.009) [9,70]. Previous studies have reported increased miR-215 expression in obesity, with normalization following substantial weight loss after bariatric surgery, suggesting a role in obesity-associated metabolic and inflammatory pathways [56]. Its increased expression in the study cohort may therefore reflect metabolic dysregulation accompanying sarcopenic obesity. miR-10b-5p is involved in adipogenesis, lipid metabolism, and cellular differentiation and has been implicated in the regulation of adipose tissue function and metabolic homeostasis [71,72]. Altered expression of miR-10b-5p has been associated with obesity-related metabolic alterations, supporting its potential relevance as a biomarker candidate of metabolic dysregulation [70,71,72].

In contrast, miR-451a (log_2_FC = 2.92; *p* = 0.009) was significantly upregulated in the present cohort. This miRNA is highly expressed in erythrocytes and is commonly considered a marker of hemolysis [9,22,29,73,74,75,76,77]. However, miR-451a has also been implicated in the regulation of the AMPK pathway, oxidative stress responses, cellular energy homeostasis, and metabolic adaptation [78]. Altered circulating levels have been reported in several metabolic and inflammatory conditions, suggesting a potential role as a biomarker of metabolic stress [9,22,70,78,79].

Because miR-451a is highly enriched in erythrocytes, even minimal degrees of hemolysis may substantially influence its measured concentration. Although no visible hemolysis was observed and identical preanalytical procedures were applied to both study groups, a dedicated hemolysis index (e.g., spectrophotometric hemoglobin assessment) was not available for this pilot dataset. Consequently, subtle hemolysis-related interference cannot be formally excluded.

Therefore, the observed increase in miR-451a should be interpreted with caution. Future validation studies should incorporate a standardized hemolysis assessment to confirm whether the observed upregulation reflects genuine pathophysiological alterations associated with sarcopenic obesity. If confirmed under conditions excluding hemolysis, miR-451a may represent a biologically relevant biomarker candidate reflecting metabolic stress, impaired energy homeostasis, and altered metabolic adaptation in sarcopenic obesity.

In addition to significantly deregulated miRNAs, several candidate miRNAs demonstrated biologically relevant fold changes despite not reaching statistical significance, including miR-29a-3p (*p* = 0.089, log_2_FC 1.89), miR-224-5p (*p* = 0.059, log_2_FC 2.35), and miR-491-3p (*p* = 0.314, log_2_FC −2.21). Although these findings should be interpreted cautiously due to the limited statistical power of this pilot study, the magnitude and direction of expression changes may indicate potential biological relevance. These miRNAs may therefore represent additional candidates for validation in larger cohorts.

### 4.2. Integrated Pathophysiological Interpretation

Collectively, the combination of muscle-enriched miR-486-5p, vascular miR-145-5p, and hepatic miR-122-5p may offer complementary insight into muscle status, vascular function, and hepatic lipid metabolism. When analyzed in combination with myogenic and metabolic markers, this miRNA panel could help distinguish patients with sarcopenic obesity from those with uncomplicated obesity or normal weight.

### 4.3. Biological Significance of the Observed Pattern

The results of this study indicate that a pattern characterized by decreased muscle-supporting and vasoprotective miRNAs together with increased hepatic and pro-metabolic stress miRNAs may reflect the coexistence of muscle atrophy, ectopic fat deposition, and dysregulated hepatic metabolic pathways, all of which are central features of sarcopenic obesity and are regulated, at least in part, by miRNAs.

Although the sample size of this pilot study was limited and the statistical power modest, the consistent trends observed across functionally related miRNAs support their potential biological relevance.

A particularly noteworthy finding in this study is the apparent paradox in absolute versus relative muscle mass between the two groups. Despite markedly elevated adiposity, the severe obesity group demonstrated a higher mean ALMI (11.38 ± 1.72 kg/m^2^) compared with the healthy controls (6.90 ± 0.93 kg/m^2^), whose DXA-derived ALMI values fell within physiologically normal ranges for young White adults according to NHANES reference data [11]. This seemingly paradoxical finding reflects a well-recognized phenomenon in severe obesity: absolute skeletal muscle mass tends to remain relatively preserved—or even elevated—as a biomechanical adaptation to the chronic mechanical load imposed by excess body weight [7]. Consequently, height-adjusted indices such as ALMI systematically overestimate muscle adequacy in severely obese individuals and are insufficient to identify sarcopenic obesity in this population.

In contrast, the ALM/W ratio—which expresses appendicular lean mass as a proportion of total body weight—was markedly lower in the severe obesity group (22.0 ± 1.8%) compared with healthy controls (31.6 ± 3.5%), whose values were well above the ESPEN/EASO 2022 sex-specific cut-offs for reduced muscle mass (<23.47% in women, <28.27% in men [7,11]). This inter-group difference in ALM/W, combined with the absence of a parallel difference in ALMI, provides direct empirical support for the methodological approach adopted in the present study: the application of the ESPEN/EASO 2022 diagnostic framework based on body weight-adjusted muscle mass rather than height-adjusted indices. The data thus illustrate why conventional EWGSOP2-based ALMI criteria would have failed to identify muscle mass deficits in this cohort, and why the ESPEN/EASO 2022 consensus was specifically designed to address this diagnostic gap in the context of severe obesity.

The present work should therefore be viewed primarily as a hypothesis-generating screening study that enabled narrowing a broad panel of candidate miRNAs to a smaller subset suitable for further validation.

### 4.4. Limitations

The limitations of this study include a relatively small sample size, the exploratory nature of the analysis, and the absence of correction for multiple testing.

Regarding the diagnostic classification of sarcopenic obesity, all patients were diagnosed according to the ESPEN/EASO 2022 consensus criteria using ALM/W as the primary body composition index. It should be noted that none of the patients fulfilled the EWGSOP2 absolute handgrip strength cut-offs for low muscle strength (<16 kg in women, <27 kg in men); in the context of severe obesity, absolute grip strength may be relatively preserved due to the mechanical demands of carrying excess body weight, potentially masking functional muscle impairment relative to lean mass. The ESPEN/EASO 2022 framework specifically recommends body weight-adjusted muscle mass indices for this population [12]. Nevertheless, the absence of confirmed low muscle strength by conventional EWGSOP2 criteria should be considered a limitation when interpreting the miRNA profiles reported here.

However, an important strength of this study lies in the comprehensive screening design, which enabled the simultaneous analysis of a large number of circulating miRNAs. This unbiased, high-throughput approach increases the likelihood of identifying novel and biologically relevant candidates that may not be captured by targeted analyses. Therefore, despite the limited sample size, the study provides valuable exploratory insights and a robust basis for subsequent validation studies.

Although Welch’s *t*-test was used for group comparisons, the results should be interpreted as hypothesis-generating rather than confirmatory. In addition, the cross-sectional design does not allow causal relationships to be established. Nevertheless, this work provides an important foundation for future research and offers valuable insights for the formulation of subsequent hypotheses. Future studies involving larger, well-characterized cohorts and longitudinal designs will be essential to further clarify the clinical relevance of the identified miRNA signatures.

## 5. Conclusions

This pilot study confirmed that circulating microRNAs may represent promising biomarkers of sarcopenic obesity. We identified specific changes in miRNA expression that reflect the interplay between muscle, adipose, and hepatic metabolic pathways, suggesting their involvement in the pathogenesis of the disease (see Table 3). Decreased expression of miR-30c-5p and miR-182-5p, together with increased levels of hepatic and inflammatory miR-122-5p and nonspecific markers of metabolic burden (miR-1290, miR-1246), may constitute a characteristic profile of patients with sarcopenic obesity. Likewise, elevated levels of the myogenic miR-486-5p and reduced expression of miR-145-5p highlight the complex involvement of pathways regulating muscle growth, endothelial/smooth muscle function, and metabolic homeostasis.

Overall, the findings of this study support the hypothesis that sarcopenic obesity results from dysregulation across multiple biological systems, including muscle metabolism, hepatic function, and chronic inflammation. Despite the limited sample size and the need to interpret *p*-values as indicative, these data represent an important first step toward validating selected miRNAs in larger cohorts. Future studies should include broader analyses in more extensive populations, incorporate functional correlations with muscle mass, strength, and metabolic parameters, and evaluate dynamic changes in miRNA expression in response to therapeutic interventions such as targeted exercise or bariatric surgery.

The targeted integration of miRNAs into clinical practice could enable earlier diagnosis, prediction of disease progression, and individualized treatment of patients with sarcopenic obesity, representing a potentially valuable direction for future research in personalized medicine, pending validation in larger, well-characterized cohorts.

## Figures and Tables

**Table 1:** Baseline characteristics of study participants.

Parameter	Healthy Controls (*n* = 6)	Severe Obesity Group (*n* = 6)
Female/male (*n*)	4/2	4/2
Age, mean ± SD (years)	27.8 ± 4.7	42.0 ± 5.4
BMI, mean ± SD (kg/m^2^)	21.0 ± 1.6	51.1 ± 8.8
BMI range (kg/m^2^)	18.9–23.6	45.1–67.6
Handgrip strength, mean ± SD (kg)	37.0 ± 8.0	32.1 ± 15.8
Total body fat, mean ± SD (%)	24.6 ± 5.8	49.4 ± 4.6
Total body fat, range (%)	16.2–31.7	43.8–56.7
VAT area (cm^2^) mean ± SD	35.8 ± 14.1	271 ± 122
ALMI (kg/m^2^), mean ± SD	6.90 ± 0.93	11.38 ± 1.72
ALMI range (kg/m^2^)	5.80–7.87	9.94–14.50
ALM/W (%), mean ± SD	31.6 ± 3.5	22.0 ± 1.8
ALM/W range (%)	26.7–35.5	20.4–24.4

Note: According to the ESPEN/EASO 2022 criteria for sarcopenic obesity (ALM/W < 23.47% for women and <28.27% for men in combination with excess adiposity), 5 out of 6 participants (83.3%) in the severe obesity group fulfilled the diagnostic criteria for sarcopenic obesity. One participant demonstrated borderline ALM/W values slightly above the recommended cut-off for women (24.1% vs. 23.47%). Abbreviations: ALM, appendicular lean mass; ALMI, appendicular lean mass index; ALM/W, appendicular lean mass-to-weight ratio; VAT, visceral adipose tissue; SD, standard deviation.

**Table 2:** Candidate circulating miRNAs meeting the predefined biological relevance threshold (|log_2_FC| > 1.5) in patients with sarcopenic obesity compared with healthy controls.

miRNA ID	Controls (*n* = 6), Mean ± SD	SO Patients (*n* = 6), Mean ± SD	Fold Change (SO vs. Controls), log_2_	*p*-Value	Mean Cq	Valid Samples, *n* (SO/Controls)
**hsa-miR-491-3p**	5.54 × 10^−10^ ±1.67 × 10^−8^	1.20 × 10^−10^ ±7.15 × 10^−11^	−2.21	0.314	32.0	6/4
**hsa-miR-142-3p**	2.64 × 10^−7^ ±5.92 × 10^−7^	6.92 × 10^−8^ ±3.07 × 10^−7^	−1.93	0.223	23.1	5/6
**hsa-miR-381-3p**	4.53 × 10^−10^ ±9.18 × 10^−9^	1.30 × 10^−10^ ±1.27 × 10^−10^	−1.80	0.527	32.0	6/5
**hsa-miR-125a-5p**	6.01 × 10^−9^ ±3.15 × 10^−8^	1.79 × 10^−9^ ±1.36 × 10^−8^	−1.75	0.342	28.8	5/6
**hsa-miR-1-3p**	6.87 × 10^−9^ ±1.42 × 10^−8^	2.08 × 10^−9^ ±7.08 × 10^−9^	−1.73	0.319	28.5	5/6
**hsa-miR-28-5p**	1.20 × 10^−8^ ±2.30 × 10^−8^	3.75 × 10^−9^ ±1.01 × 10^−8^	−1.69	0.319	27.5	5/6
**hsa-miR-130a-3p**	1.01 × 10^−7^ ±4.15 × 10^−7^	3.17 × 10^−8^ ±2.62 × 10^−7^	−1.67	0.660	24.2	5/6
**hsa-miR-30c-5p**	1.81 × 10^−7^ ±1.15 × 10^−7^	5.99 × 10^−8^ ±3.55 × 10^−8^	−1.60	0.046 *	24.2	6/6
**hsa-miR-145-5p**	4.42 × 10^−8^ ±2.50 × 10^−8^	1.52 × 10^−8^ ±6.85 × 10^−9^	−1.54	0.027 *	25.2	6/6
**hsa-miR-182-5p**	1.07 × 10^−9^ ±3.59 × 10^−10^	3.75 × 10^−10^ ±1.24 × 10^−10^	−1.51	0.004 *	31.8	6/5
**hsa-miR-194-5p**	4.31 × 10^−10^ ±3.73 × 10^−10^	1.25 × 10^−9^ ±1.92 × 10^−9^	1.54	0.156	30.4	5/6
**hsa-miR-330-3p**	1.61 × 10^−9^ ±6.14 × 10^−9^	4.99 × 10^−9^ ±2.13 × 10^−9^	1.63	0.863	28.9	5/4
**hsa-miR-34c-5p**	1.50 × 10^−10^ ±4.68 × 10^−11^	5.08 × 10^−10^ ±1.81 × 10^−9^	1.76	0.383	31.9	5/3
**hsa-miR-486-5p**	5.50 × 10^−8^ ±1.61 × 10^−8^	1.92 × 10^−7^ ±1.06 × 10^−7^	1.80	0.016 *	23.2	6/6
**hsa-miR-17-3p**	1.92 × 10^−9^ ±4.12 × 10^−9^	7.09 × 10^−9^ ±8.18 × 10^−9^	1.89	0.303	28.8	4/2
**hsa-miR-29a-3p**	1.95 × 10^−8^ ±3.56 × 10^−8^	7.24 × 10^−8^ ±1.73 × 10^−8^	1.89	0.089	24.7	6/6
**hsa-miR-215-5p**	5.20 × 10^−10^ ±1.94 × 10^−10^	2.14 × 10^−9^ ±9.71 × 10^−10^	2.04	0.003 *	29.8	6/6
**hsa-miR-10b-5p**	4.65 × 10^−10^ ±1.01 × 10^−10^	2.08 × 10^−9^ ±1.14 × 10^−9^	2.16	0.011 *	29.9	6/6
**hsa-miR-224-5p**	1.93 × 10^−9^ ±5.25 × 10^−9^	9.84 × 10^−9^ ±5.71 × 10^−9^	2.35	0.059	24.4	6/6
**hsa-miR-885-5p**	3.00 × 10^−10^ ±2.38 × 10^−10^	1.78 × 10^−9^ ±1.61 × 10^−9^	2.57	0.040 *	30.4	6/6
**hsa-miR-451a**	1.68 × 10^−6^ ±4.56 × 10^−7^	1.27 × 10^−5^ ±7.25 × 10^−6^	2.92	0.009 *	17.7	6/6
**hsa-miR-144-3p**	7.09 × 10^−9^ ±7.32 × 10^−9^	5.53 × 10^−8^ ±2.56 × 10^−8^	2.96	0.012 *	25.6	6/6
**hsa-miR-106a-5p**	1.32 × 10^−8^ ±1.12 × 10^−7^	1.12 × 10^−7^ ±4.62 × 10^−8^	3.08	0.248	26.2	5/5
**hsa-miR-1290**	1.54 × 10^−8^ ±6.74 × 10^−9^	1.54 × 10^−7^ ±8.91 × 10^−8^	3.32	0.009 *	24.3	6/6
**hsa-miR-193a-5p**	1.71 × 10^−10^ ±5.71 × 10^−11^	1.81 × 10^−9^ ±1.92 × 10^−9^	3.40	0.036 *	30.7	6/6
**hsa-miR-122-5p**	2.64 × 10^−9^ ±2.22 × 10^−9^	4.96 × 10^−8^ ±4.26 × 10^−8^	4.23	0.025 *	26.4	6/6
**hsa-miR-1246**	1.01 × 10^−9^ ±4.72 × 10^−9^	2.12 × 10^−8^ ±3.90 × 10^−8^	4.39	0.044 *	27.6	6/6

Note: * Indicates candidate miRNAs with statistically significant differential expression between patients with sarcopenic obesity and healthy controls (*p* < 0.05).

**Table 3:** Overview of the identified miRNAs, their known biological functions, and direction of expression in the study.

miRNA	Main Biological Function	Relevance to Sarcopenic Obesity (SO)	Direction of Expression in the Study
miR-486-5p	Myogenesis, activation of the IGF-1/PI3K/AKT pathway, inhibition of PTEN/FOXO1A	Supports muscle growth and regeneration; increased after physical activity and adaptation	↑ increased
miR-145-5p	Differentiation of smooth muscle cells, vascular homeostasis	Decrease associated with endothelial dysfunction and impaired muscle perfusion	↓ decreased
miR-122-5p	Hepatic marker, regulation of lipid metabolism (NAFLD, dyslipidemia)	Increased in obesity and hepatic burden	↑ increased
miR-30c-5p	Regulation of lipid metabolism	Triglyceride accumulation, dyslipidemia	↓ decreased
miR-182-5p	Cell proliferation, stress response, apoptosis	Impaired muscle regeneration and increased cellular vulnerability	↓ decreased
miR-1290	Insulin resistance, inflammation, metabolic stress	Increased in obesity and diabetes; potential marker of metabolic dysfunction and insulin resistance	↑ increased
miR-1246	Exosomal signaling, adipose tissue homeostasis, macrophage polarization, metabolic regulation	Associated with metabolic stress and systemic inflammation; biological relevance in obesity-related disorders remains to be clarified	↑ increased
miR-193a-5p	Metabolic regulation, NAFLD progression, diagnostic biomarker potential	Potential marker of metabolic dysregulation and chronic low-grade inflammation	↑ increased
miR-885-5p	Cellular metabolism, inflammation	Possible link with metabolic stress and immune regulation	↑ increased
miR-144-3p	Lipid metabolism, oxidative stress	Connection with inflammation and lipid/glucose metabolism	↑ increased
miR-215-5p	Cell proliferation, differentiation, metabolic regulation	Potentially associated with glucose metabolism and metabolic dysregulation	↑ increased
miR-10b-5p	Adipogenesis, lipid metabolism, adipocyte differentiation	Obesity-related metabolic dysregulation and adipose tissue dysfunction	↑ increased
miR-451a	AMPK signaling, metabolic adaptation, oxidative stress response, erythrocyte-enriched miRNA	Potential marker of metabolic stress; interpretation limited by possible hemolysis	↑ increased (interpretation requires consideration of hemolysis)

## Data Availability

All datasets generated and analyzed during this study are provided in the Supplementary Materials, including raw Cq values and processed expression data for all analyzed miRNAs.

## Supplementary Materials

The following supporting information can be downloaded at: https://www.mdpi.com/article/10.3390/biomedicines14061377/s1. Table S1. Primary raw Cq data for all analyzed samples; Table S2. Processed miRNA expression dataset including normalized expression values, fold changes, and statistical parameters; Table S3. Shapiro–Wilk normality test results for candidate miRNAs.

## Abbreviations

The following abbreviations are used in this manuscript:

ALM	Appendicular lean mass
ALMI	Appendicular lean mass index
ALM/W	Appendicular lean mass to body weight ratio
AMPK	AMP-activated protein kinase
BIA	Bioelectrical impedance analysis
BMI	Body mass index
Cq	Quantification cycle
DXA	Dual-energy X-ray absorptiometry
EASO	European Association for the Study of Obesity
EK	Ethics Committee
EWGSOP2	European Working Group on Sarcopenia in Older People 2
ESPEN	European Society for Clinical Nutrition and Metabolism
miRNA	MicroRNA
NAFLD	Non-alcoholic fatty liver disease
PCR	Polymerase chain reaction
RT-qPCR	Reverse transcription quantitative polymerase chain reaction
SD	Standard deviation
SO	Sarcopenic obesity
